# The ATOM-Seq sequence capture panel can accurately predict microsatellite instability status in formalin-fixed tumour samples, alongside routine gene mutation testing

**DOI:** 10.1038/s41598-024-72419-7

**Published:** 2024-09-19

**Authors:** Kanishta Srihar, Arief Gusnanto, Susan D. Richman, Nicholas P. West, Leanne Galvin, Daniel Bottomley, Gemma Hemmings, Amy Glover, Subaashini Natarajan, Rebecca Miller, Sameira Arif, Hannah Rossington, Thomas L. Dunwell, Simon C. Dailey, Gracielle Fontarum, Agnes George, Winnie Wu, Phil Quirke, Henry M. Wood

**Affiliations:** 1 Pathology and Data Analytics, Leeds Institute of Medical Research at St James’s, University of Leeds, Leeds, UK; 2 Department of Statistics, University of Leeds, Leeds, UK; 3 Leeds Institute of Data Analytics, University of Leeds, Leeds, UK; 4 GeneFirst Ltd., Abingdon, UK; 5 NIHR Leeds Biomedical Research Centre, Leeds, UK

**Keywords:** Microsatellite instability, Gene panel, Colorectal cancer, Mismatch repair, Prognostic markers, Gastrointestinal cancer, Tumour biomarkers

## Abstract

Microsatellite instability (MSI) occurs across a number of cancers and is associated with different clinical characteristics when compared to microsatellite stable (MSS) cancers. As MSI cancers have different characteristics, routine MSI testing is now recommended for a number of cancer types including colorectal cancer (CRC). Using gene panels for sequencing of known cancer mutations is routinely performed to guide treatment decisions. By adding a number of MSI regions to a small gene panel, the efficacy of simultaneous MSI detection in a series of CRCs was tested. Tumour DNA from formalin-fixed, paraffin-embedded (FFPE) tumours was sequenced using a 23-gene panel kit (ATOM-Seq) provided by GeneFirst. The mismatch repair (MMR) status was obtained for each patient from their routine pathology reports, and compared to MSI predictions from the sequencing data. By testing 29 microsatellite regions in 335 samples the MSI status was correctly classified in 314/319 samples (98.4% concordance), with sixteen failures. By reducing the number of regions in silico, comparable performance could be reached with as few as eight MSI marker positions. This test represents a quick, and accurate means of determining MSI status in FFPE CRC samples, as part of a routine gene mutation assay, and can easily be incorporated into a research or diagnostic setting. This could replace separate mutation and MSI tests with no loss of accuracy, thus improving testing efficiency.

## Introduction

Deficient mismatch repair (dMMR) and the associated microsatellite instability (MSI) represent a subset of cancers with an association with Lynch syndrome and different clinical characteristics, outcomes and response to chemotherapy and immunotherapy when compared to proficient mismatch repair, microsatellite stable (pMMR/MSS) tumours in similar anatomical sites. This is particularly relevant for colorectal cancer (CRC), where dMMR/MSI is seen in approximately 12–17% of tumours, and is associated with older age, right-sidedness, better prognosis in intermediate stage disease, poorer response to standard chemotherapy and better response to immunotherapy^1^. Approximately 3% of CRC are associated with Lynch syndrome, which is caused by a germline mutation in the MMR genes and results in dMMR/MSI tumours, generally in younger patients^2^. In the UK, routine MMR/MSI testing of all CRC at diagnosis has been mandated by NICE since 2017 primarily to screen for Lynch syndrome, however the results are routinely used to guide neoadjuvant and adjuvant decisions, so quick and accurate detection is becoming increasingly important^3^.

Detection of dMMR is traditionally performed using immunohistochemistry (IHC) for four proteins; hMLH1, hMSH2, hMSH6 and PMS2, looking for loss of expression of one or more proteins in tumour cells. Alternatively, MSI has been directly measured using PCR-based approaches, where fragment length analysis investigates increased variation in repeat lengths of the microsatellite regions^4^. Using enough markers in this way allows greater resolution between simple MSI/MSS, with MSI-high and MSI-low subtypes also being defined^5^. However, uptake of dMMR/MSI testing has been slow. A survey by Bowel Cancer UK in 2018 showed that only 18% of centres were following the NICE guidance^6^. The 2022 National Bowel Cancer Audit (NBOCA) report showed that reporting of MMR/MSI status had improved from 13% to only 21% between 2018/19 and 2020/21^7^, although this had increased to over 90% by 2024^8^. Gene panel testing is commonly performed for CRC patients with metastatic disease to guide the use of targeted therapies. Older sequencing technologies have largely been replaced by next-generation sequencing (NGS), through which assessing the MSI status from genomic sequence data is possible^9–12^. Approaches typically identify a number of potential microsatellite repeats from the regions sequenced, then collect information about the distribution of repeat lengths of those regions^9,11,12^. If a region has a length distribution more variable than either a matched control or pool of known MSS samples, it is deemed to have mutated. If enough regions have mutated (typically 20%), then the sample is predicted to be MSI.

While some of these approaches require exome or whole genome sequence data, some have been shown to work on targeted sequence panels with a limited number of repeat regions^10^. Adding a small number of MSI regions to a limited gene panel is an inexpensive way to gather more clinically relevant information about a patient without needing to do separate dMMR/MSI testing.

One such approach is the GeneFirst ATOM-Seq targeted capture panel^13^. This is a new approach, based on single target primer PCR of targeted regions, designed to work with clinical samples, such as formalin-fixed paraffin-embedded (FFPE) tumour blocks. It incorporates unique molecular identifiers (UMIs) directly to the 3’ ends of each template molecule to allow for efficient PCR duplication removal, ensuring that the final sequence data represents the original template. This can be done alongside testing for other known mutation hotspots. As part of a larger study to evaluate the ATOM-Seq technology, its ability to determine MSI status in a cohort of FFPE CRC tumours was tested using samples from consented patients recruited into the Yorkshire Cancer Research Bowel Cancer Improvement Programme (YCRBCIP).

This study is designed to answer current limitations in the literature where only a small number of studies exist. These are: whether a combined test such as this, with mutations and MSI testing, can be performed in a clinically meaningful period and therefore be useful in pre-operative testing; where there are disagreements between MSI and MMR testing, could a large panel of MSI regions such as this prove useful in determining the true status.

## Results

### Sample numbers and sequencing metrics

Sequencing was performed on 335 FFPE CRCs with previously determined dMMR/MSI status. Of these, 47 (14%) were dMMR/MSI, with 288 (86%) being pMMR/MSS. After using UMI sequences to merge PCR duplicates, mean sequencing coverage across the entire panel region ranged from 0.01× to 4126× coverage (mean 950×, median 853×). Poorly performing samples were not removed at this stage, in order to test the lower limits of MSI detection capacity. A sample with a mean coverage of 1× might have better coverage in one or more of the MSI regions.

### Main findings: MSI testing with optimal conditions

MSI calling was assessed using three different approaches: mSINGSlw^14^, MSIsensor-pro^11^ and DeltaMSI^12^.

**Fig. 1:**
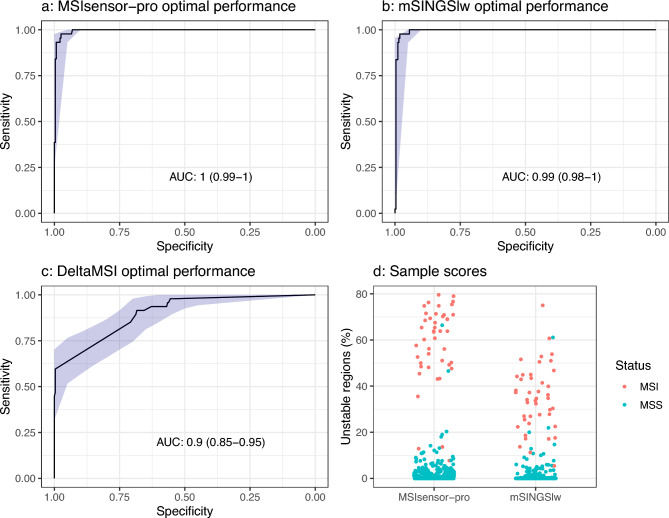
Assay performance under optimal conditions. (**A**–**C**) show the cross-validation ROC curves for MSI-sensor-pro, mSINGSlw and DeltaMSI, using 111–112 training samples and all 29 genomic regions. (**D**) shows the range of individual sample scores for MSIsensor-pro and mSINGSlw, coloured by known dMMR/MSI status.

After cross validation using one third of the samples as a training set in each case, and all regions of the sequencing panel, mSINGSlw mis-called six samples with nineteen failing due to lack of sequence depth. MSIsensor-pro mis-called five samples with sixteen samples failing (Fig. 1A–C). A summary of the results is shown in Table 1, with full details in Supplementary Table S1.

**Table 1:** Samples, mis-calls, area under the curve (AUC) and failures for the three tested algorithms under optimal conditions. False positive refers to samples previously called pMMR/MSS which were called MSI here. False negative is the inverse.

Algorithm	mSINGSlw	MSIsensor-pro	DeltaMSI
Samples	335	335	335
False positives (%)	3 (1%)	2 (0.7%)	2 (0.7%)
False negatives (%)	3 (6.4%)	3 (6.4%)	18 (38.3%)
AUC	0.99	1	0.92
Failures (%)	19 (5.7%)	16 (4.5%)	0

One MSI sample and one MSS sample were mis-called by both algorithms. All the samples which failed with MSIsensor-pro also failed with mSINGSlw. Examining the individual scores for each sample, the MSI and MSS samples separated more clearly for MSIsensor-pro, but it was still possible to identify a sensible cut-off for mSINGSlw (Fig. 1D).

Delta-MSI only used two or three of the 29 available regions in most cases. This gave a range of scores too limited to define sensible cutoffs, and an area under the curve (AUC) of 0.92. As it was not performing as well as the other two algorithms, it was not tested further.

For individual replicates within the cross-validation, mSINGSlw had AUCs ranging from 0.97 to 1. The failure rate was 3.6–8%. MSIsensor-pro had AUCs ranging from 0.97 to 1. The failure rate was 3.1–6.7%.

### Additional findings: MSI testing under sub-optimal conditions

Whilst the performance of the assay was very good, it is conceivable that future uses of this or similar assays might have fewer samples in their training sets, or would wish to use a smaller panel of MSI regions. As such the remainder of the results section details how the assay was tested using reduced sample and assay sizes to explore the limits at which the assay could be used.

### Reducing training set and number of regions

**Fig. 2:**
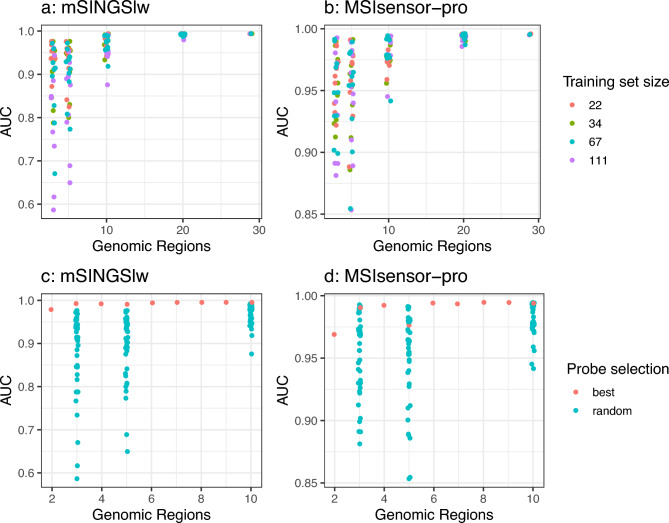
The effect of reducing the size of the training set and number of regions examined. (**A**) and (**B**) show the effect on AUC of reducing the number of genomic regions examined at random, and reducing the size of the training set. (**C**) and (**D**) show the difference between random and optimal selection between 2 and 10 genomic regions.

Cross validations were repeated after randomly sub-sampling the regions examined, and reducing the size of training set. (Fig. 2A,B). For 20 and 29 regions, the choice and size of training samples had negligible effect. For smaller subsets of regions (3, 5 and 10) there was more variation. MSIsensor-pro appeared more resilient to smaller region numbers. Replicates with three regions had AUCs ranging from 0.59 to 0.98 for mSINGSlw and from 0.88 to 0.99 for MSIsensor. Using five regions gave AUCs ranging from 0.65 to 0.98 for mSINGSlw and 0.85–0.99 for MSIsensor-pro. Using ten regions gave AUCs ranging from 0.88 to 0.99 for mSINGSlw and 0.94–0.99 for MSIsensor-pro. Variation in AUC appeared random within region size and completely independent of the size of training set.

### Finding optimal sets of regions

Using a backwards/forwards approach to define optimal genomic regions (Fig. 2C,D), mSINGSlw performed better than when using any randomly selected combination of regions. Using three regions was enough to achieve an AUC of over 0.99, but had a failure rate of 11.0%. Failure rate reached the minimal level of 4.7% with seven regions.

MSIsensor-pro using selected combinations of regions performed better than most but not all random combinations. Some randomly selected combination of regions and training sets performed better than the optimally selected region combination due to frequently mis-called samples being either randomly present or absent in that combination of validation samples. Optimal performance was reached with eight regions, when the failure rate reached 3.6%.

### Examining lower depth thresholds

Both algorithms were repeated with depth cutoffs of 30, 20, 15 and 10× coverage to accept a region for processing. The AUCs were unchanged from the original cross-validations in all cases. False positive and false negative rates were very similar. mSINGSlw mis-classified one fewer sample with a threshold of 10× coverage, while MSIsensor-pro mis-classified one extra sample with 10× coverage. As the coverage threshold decreased, the number of samples failing reduced from 19 to 13.

### Exploring mis-called samples

**Fig. 3:**
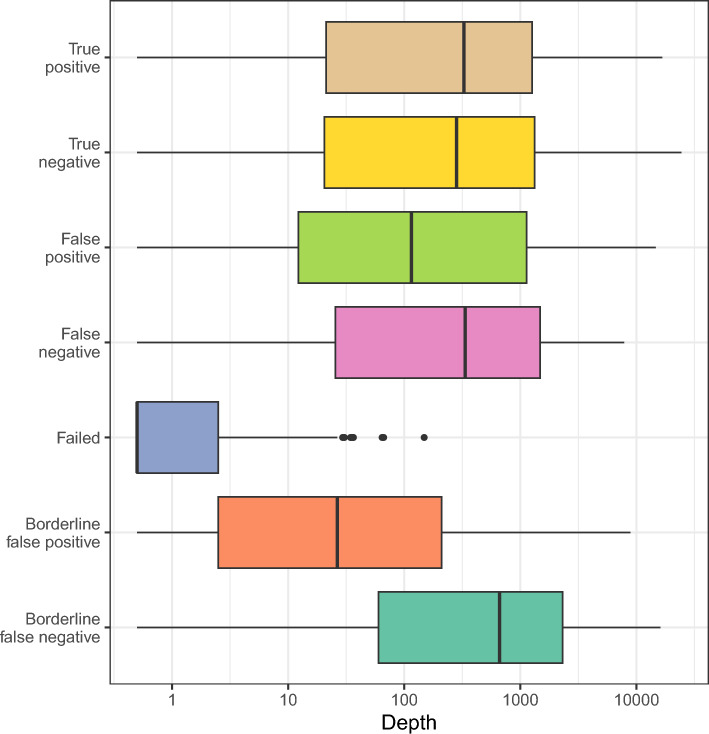
The range of sequence depths across all regions. Samples are split into those that were called correctly, mis-called, failed, or were correct but borderline.

The coverage for all regions across all samples was examined, counting how many reads contained the repeat and flanking sequence, so were able to be considered for analysis (Fig. 3). For correctly called samples, and false negatives, the distribution of depths was the same. For failing samples, the depths were consistently low, with only 1% of regions having a depth of over 30 (correctly called samples had a rate of 73%). For false positive samples, 66% regions had a depth over 30. For borderline false positives (samples correctly called, but close to the threshold) this value was 47%. This indicates that lower sequence depth was linked to false positive calls. However, false negative calls, and borderline false negatives were associated with higher depth.

To test whether the association of lower depth with false positives was just due to smaller number of regions available for testing in low-depth samples, or due to lower depth regions giving a smaller sample of repeat length distributions, each sample was subsampled to a series of reduced depths. Sequences were randomly sampled for each region up to a maximum of 40, 60 and 80× coverage, so that no extra regions would fail, but that the amount of data would be reduced. This had no effect at all on mis-call rate or AUC, indicating that low depth samples were more likely to be false positives due to fewer regions passing filters.

To examine if discrepancies could be due to low tumour cell content, all nine discrepant samples were examined. Their tumour cell contents were ranged from 30 to 70%, all high enough to disregard the possibility of low tumour cell content leading to mis-calling.

To understand if initial mis-calls of MMR status could be responsible for discrepancies, the nine discrepant samples were re-stained in our University laboratory and re-called by a Consultant GI Pathologist. Six had unchanged status. One sample which was called MSI by MSIsensor-pro and MSS by mSINGSlw was initially called dMMR and re-called as pMMR. The two samples which were supposedly mis-called by both algorithms were re-called differently (one from dMMR to pMMR and one from pMMR to dMMR). If the new MMR calls were taken to be correct, MSIsensor-pro would move from five discrepant samples to four, while mSINGSlw would move from six discrepant samples to three.

## Discussion

This study sought to examine the efficacy of MSI testing in a series of FFPE CRC samples using a limited gene panel containing 29 microsatellite regions. Results were compared to the gold standard IHC data or PCR testing. Two popular MSI calling algorithms were used under default settings. Both approaches gave ROC AUCs of between 0.99 and 1, suggesting that incorporating a limited number of repeat regions into a gene panel is an effective way identifying MSI, and is a very attractive option for adding MSI information to routine gene testing. By combining MSI testing with gene mutation analysis in one assay, as opposed to separate testing, time and costs are saved, and precious sample material conserved. This works in FFPE material, so could easily be introduced into a diagnostic or research setting. By combining MSI testing with *BRAF* V600E mutation calling, testing for Lynch syndrome can be performed with a single assay. MSS samples can be examined for mutations in genes such as *KRAS*, NRAS or *PIK3CA*.

There was extremely high concordance with the MSI/dMMR data obtained from pathology reports. Depending on the algorithm used, only five or six (1.4–1.8%) out of 335 samples were mis-called, with either 16 (4.5%) or 19 (5.7%) samples not being assessed due to limited sequenced depth. The gold standard dMMR IHC assay is also not perfect and can both miss and overcall dMMR. Published estimates of sensitivity range between 85.7 and 100% and specificity between 80.5 and 91.9%, depending on the prevalence in the population tested^15–17^.

Examining the samples mis-called in detail, only one putative MSS sample was mis-called by both algorithms. Examining the gene mutation data from this sample showed that it had more mutations called than any of the other samples, which suggests it may have had a different hypermutator phenotype, such as that caused by a *POLE* mutation^18^. This may cause microsatellites to be mutated, whilst having no effect on IHC of the mismatch repair proteins. However, restaining this sample gave a pMMR status, which also indicates a possible pathology error in the initial MMR calling.

One putative MSI sample was mis-called by both algorithms. Sequence depth for this sample was well above thresholds. It was not close enough to the cut-offs to be a borderline call, although it was above average for genuine MSS samples. Upon restaining, this sample was called as dMMR, indicating either a sample that was difficult to call, or a pathology error in the initial MMR calling.

Of the remaining mis-called samples, all had either poor depth, too few regions available for comparison, and/or were close to the cut-off thresholds. It is also likely that some iterations of the cross validations had more than usual numbers of borderline or failing samples in the training cohort, which would reduce performance of the remaining borderline cases in the test cohort. Assay performance did not appear to be linked to tumour cell content, with all discrepant cases showing moderate or high levels.

The small number of discrepancies and borderline correctly called cases close to the cut-offs does not indicate any weakness in this assay. It is reported that MSI and dMMR concordance in only around 93% with standard testing methods^4^ and that dMMR matches the MSI-high genotype (defined as > 30% of markers mutated)^5^. MSI-low samples have clinical features similar to MSS, but have some mutations in repeat regions, once enough are examined. Our concordance levels are 98% by either methodology, suggesting that an NGS approach such as this outperforms standard MSI testing, and that MSI and dMMR are actually more associated than previously thought. Using enough markers in a gene panel allows MSI-low cases to be observed, which account for most of the apparent discrepancies.

The assay failure rate is comparable to other methods of MSI testing: 4.2% of samples, which is comparable to the 3.4% reported for analysis of FFPE samples with the Idylla MSI testing system^19^. There was no upstream quality filtering of samples. Tissue blocks were simply processed as they arrived from their respective hospitals.

This assay used 29 repeat regions, with that number being chosen as to be small enough to add to a limited gene panel, but large enough to cover assay failure in some regions. However, by carefully selection the optimal combinations, equivalent performance with only eight regions was possible.

This is comparable to a recent study using a dedicated MSI panel (plus *BRAF*) where the performance of the full panel of 24 markers could be replicated using only six^10^. As with that study, using a limited panel risked the robustness of the assay, as samples with depth close to the threshold might fail in the selected regions, but have enough sequence to call when the entire panel was used. Using a very limited number of regions would also reduce the ability to detect some MSI-low samples, or distinguish others from MSI-high. This assay suffered only occasionally lower performance with all combinations of 20 randomly selected regions, so it is likely that selected combinations of between 15 and 20 regions would give good performance and assay robustness. We have used this assay with CRC resection samples, but it could be applicable to other tumour types, biopsies, or liquid biopsies.

Although three different software packages were tested, this is not a comparison of algorithms. The aim was to quickly find two or three approaches that worked, to explore the performance of this assay, rather than replicate the comparisons found in the papers supporting each package. The three algorithms initially used were chosen due to popularity and their stated ability to work with limited markers, and no need for a matched control sample.

Delta-MSI typically only chose two or three of our 29 regions for analysis under default settings. This gave samples a limited range of scores (0, 0.33, 0.5, 0.67, 1). As such, effective cut-offs between MSS and MSI samples were difficult to produce, and the ROC curves very angular. It might be that subtle tweaks to the software parameters would have allowed a larger range of regions to be analysed, but as the other two packages were giving AUCs of over 0.99, this was not explored further.

MSIsensor-pro and mSINGSlw gave similar performance in terms of misclassifications and AUC. More samples failed with mSINGSlw, but that was due to the higher default depth cut-off (30× rather than 15×). When both algorithms used the same cut-offs, the performances were comparable. Looking at the scores for each sample, MSIsensor-pro gave better separation between MSI and MSS cases and therefore would have more repeatable performance.

These results could potentially have far-reaching clinical importance. Microsatellite testing is very important, as we know that in the pre-operative setting it is possible to obtain an 80–90% response rate with six weeks of immunotherapy in some MSI cancers which do not respond well to standard treatment^20,21^. In distant metastatic disease response rates fall but still reach 40–50% providing significant benefit. In addition, these tumours have a different clinical background within the colon, tending to be right sided in older females or Lynch patients which has significant implications for their management and their families. Routine screening of all bowel cancers for MSI and, if positive, for Lynch syndrome is recommended within many health care systems including the United Kingdom National Health Service. MSI CRC tumours also metastasise less often but may be more locally advanced than non MSI tumours. Their pattern of mutations in antigen presentation genes is also very different to non MSI tumours^22^.

This study has a number of limitations, which we have sought to minimise. By its nature, this study is limited to the testing of one assay, the GeneFirst ATOM-seq panel. This is similar to other studies reporting single NGS assays for MSI testing^10^. To make the study as generalisable as possible, multiple different panel sizes were informatically tested, and three different analysis tools were used. It is also possible that the cohort is not reflective of all CRC patients, and may have hidden confounding factors. However, by using real-world patients from multiple hospitals, as they were recruited, this study does not select by age and represents a random selection of bowel cancer patients in the UK. As such, the patient demographics are a strength of this study.

## Summary

With the increased use of small gene panels in both diagnostic and research settings, the addition of a limited number of repeat regions can allow MSI status to be easily derived with no additional tests. The GeneFirst ATOM-Seq technology is ideally suited to perform this task, and gives performance as good as, or better than comparable methods. Assays such as this could be used to improve patient care by helping to choose the most appropriate care, either minimising unnecessary treatment or switching to alternative therapies.

## Methods

### Overview

This study was part of a larger study to evaluate the ATOM-seq sequencing technology. This panel was used to sequence DNA from a cohort of CRC samples collected as part of routine care. MSI testing on the resulting sequence data was performed using three popular tools, and compared with the diagnostic reports, which were produced from either IHC or PCR-based methods.

As the tools used need a pool of known MSS and MSI samples for comparison, multiple iterations of splitting the samples into test and validation cohorts were used. Different sizes of test cohort and panel size were used in multiple cross-validations, to thoroughly test the tools in ideal and more difficult situations.

In all instances, numbers and proportions of concordant and discordant samples were measured using defined cutoffs as well as using ROC curves to examine overall performance.

### Samples and ethics

FFPE CRC tumour resection samples were collected from five hospitals across the Yorkshire and Humber region of the UK through the Yorkshire Cancer Research Bowel Cancer Improvement Programme (YCR BCIP)^23^. The first 335 patients to be recruited were analysed. Patients provided informed consent for the study.

DNA was extracted using the Qiagen QIAmp DNA Micro extraction kit. Guided by an annotated H&E slide, 10 × 5-micron sections of FFPE block were macrodissected, before dewaxing, proteinase K incubation and DNA extraction as per kit protocols.

Ethical approval was granted by the Solihull—West Midlands Ethics Committee, reference number 17/WM/0374. All methods were carried out in accordance with relevant guidelines and regulations.

### Clinical dMMR/MSI testing

Mismatch repair or MSI status was obtained from routine pathology reports. The majority of samples were tested for dMMR using IHC^24^. Three samples from one hospital were assessed using the Idylla PCR-based MSI test^19^.

### Library preparation and sequencing

A 16.8 Kb, 155 region ATOM-Seq^13^ targeted capture panel was designed to capture common and/or actionable CRC somatic mutations in 23 genes, plus 29 microsatellite targets (Supplementary Table S1). This panel was used to prepare sequencing libraries from the extracted tumour DNA.

Successfully prepared libraries were pooled to equimolar concentrations, with between 87 and 144 samples per pool. Pools were sequenced on an Illumina NextSeq 2000 P2 run, at 2 × 150 bp read lengths.

### Basic sequence processing

UMIs were added to each read based on the first 20 bp of the reverse reads. The reads were aligned to the human genome (HG38) using BWA-mem (0.7.17-r1188)^25^. The UMIs were then used to merge PCR duplicates by Gencore (0.13.0)^26^.

### MSI detection software

Three different algorithms were used to determine MSI status. mSINGSlw^14^, a lightweight implementation of mSINGS^9^, MSIsensor-pro (1.2.0)^11^ and DeltaMSI (1.0.1)^12^. All tools were used under default parameters.

Each algorithm returned a score, indicating a proportion of genomic positions thought to be unstable for each sample. Optimal cut-offs for defining MSI status were defined as those giving the smallest number of mis-called samples. Area under the curve (AUC) values were also calculated using the pROC (1.18.4)^27^ package in R.

### Cross validation, testing different sized panels, number of controls and depths

For cross validation purposes, ten replicates of test and validation samples were randomly generated, with the samples being split into three combinations of 1:2, five combinations of 1:4 or ten combinations of 1:9 test:validation ratios. So, for the 1:2 ratio, 30 replicates were performed. The mean and individual score for each sample across each combination of replicates was collected.

To test the efficacy of different sized panels, the full panel was randomly subsampled with ten replicates each of three, five, ten and twenty regions, each of which was subjected to the full set of cross validation sample combinations.

These combinations of samples and regions were pre-defined and used in all experiments, to ensure that different tools and parameters received a like-for-like comparison.

To find optimal panels of genomic regions, the 1:2 cross validation sample combinations were tested with gradually increasing sized panels using a backward-forward iterative process. Starting with one region, each region was tested to find the best region to add, before all regions in the growing panel were removed in turn to find the region the new panel needed least. This process was repeated until no improvement could be found, when the new best panel was recorded, and the number of regions increased by one. Panels were judged on the fewest mis-called samples, with fewest failing samples being used to split ties.

To test the effect of different minimum sequence depths being imposed on the different algorithms, the 1:2 cross validation sample combinations and full set of regions were tested with minimum depths to accept a region of 10, 15, 20 and 30-fold coverage.

To investigate whether performance decreased in samples close to the minimum coverage, the 2:1 cross validation was performed with all test samples subsampled to 40×, 60× and 80× coverage.

## Supplementary Information

Supplementary Tables.

## Data Availability

Raw sequence data is available from the European Nucleotide Archive, accession PRJEB74030 The mSINGSlw implementation of mSINGS is available at https://github.com/drhenrywood/mSINGSlw.
